# Web-based software applications for frailty assessment in older adults: a scoping review of current status with insights into future development

**DOI:** 10.1186/s12877-021-02660-6

**Published:** 2021-12-18

**Authors:** Riley Chang, Hilary Low, Andrew McDonald, Grace Park, Xiaowei Song

**Affiliations:** 1 Clinical Research, Surrey Memorial Hospital, Fraser Health, Critical Care Tower T2-820, 13750 96th Avenue, Surrey, BC V3V 1Z2 Canada; 2 Department of Evaluation and Research Services, Fraser Health, Surrey, BC Canada; 3 Department of Medicine, University of British Columbia, Vancouver, British Columbia Canada; 4 Department of Primary Care and Home Health, Fraser Health, Surrey, BC Canada; 5 Department of Biomedical Physiology and Kinesiology, Simon Fraser University, Burnaby, BC Canada

**Keywords:** Comprehensive geriatric assessment, Digital health, Frailty assessments, Web-based software, Web applications

## Abstract

**Background:**

A crucial aspect of continued senior care is the early detection and management of frailty. Developing reliable and secure electronic frailty assessment tools can benefit virtual appointments, a need especially relevant in the context of the COVID-19 pandemic. An emerging effort has targeted web-based software applications to improve accessibility and usage. The objectives of this scoping review are to identify and evaluate web-based frailty assessment tools currently available and to identify challenges and opportunities for future development.

**Methods:**

We conducted a review with literature (e.g., using MEDLINE databases) and Google searches (last updated on October 10, 2021). Each of the identified web applications were assessed based on eight featured categories and assigned a rating score accordingly.

**Results:**

Twelve web-based frailty assessment applications were found, chiefly provided by the USA (50%) or European countries (41%) and focused on frailty grading and outcome prediction for specific patient groups (59%). Categories that scored well among the applications included the User Interface (2.8/3) and the Cost (2.7/3). Other categories had a mean score of 1.6/3 or lower. The least developed feature was Data Saving.

**Conclusions:**

Web-based applications represent a viable option for remote frailty assessments and multidisciplinary integrated care of older adults. Despite the available web-based frailty assessments on the Internet, many missed certain needed features for professional use in healthcare settings. This situation calls for fully comprehensive web-based applications, taking into consideration a number of key functions linking graphical user interface and functionalities, and paying special attention to secure data management.

**Supplementary Information:**

The online version contains supplementary material available at 10.1186/s12877-021-02660-6.

## Background

Frailty is a multidimensional state of increased vulnerability with age that can lead to increased risk of many adverse outcomes, including hospitalization and mortality [1, 2]. Frailty can be operationalized in many ways, although none of them have provided a definite diagnosis [2–6]. The phenotypic approach categorizes the physical presentation of frailty based on five clinical features including weakness, slow walking speed, unintentional weight loss, exhaustion, and low physical activity [7, 8]. The deficit accumulation based frailty index estimates the proportional presentation of health problems of a wide range of health domains [6, 9]. Both approaches have gained popularity and been widely used in clinical and epidemiological studies [10]. Ongoing efforts are being made to enable frailty-informed care of older adults involving multidisciplinary care teams [10]. For such integrated care, early detection and management of frailty is key [10].

Emerging data have also highlighted the relationship between frailty and the COVID-19 deaths and other adverse effects in older adults, who are more likely to have comorbidities of multiple organ systems and to encounter other medical and social challenges [11–13]. Following the physical distancing guidelines, virtualized approaches are being increasingly adopted to continue senior care and frailty management, while reducing in-person appointments [14, 15]. Besides the pandemic, some older adults may also be beset by transportation related issues that prevent easy access to primary care (e.g. mobility problems, lack of caregiver ability to drive, no driver's license, remote area) [16, 17], increasing the need for reliable virtual assessments.

Web-based health assessments have potential to benefit virtual appointments and frailty detection, as they allow care providers of different settings to feasibly access assessment data and better understand the level of frailty and its changes in individual patients in developing optimized care plans (e.g. intervention strategies and discharge care). As well, the potential large-scale data sharing over the Internet can benefit demographical research in promoting the health of older adults in aging populations. In general, web-based applications can also be available for free or at a low cost, more time efficient, and allow automatic calculations leading to immediate results [18–21]. It is anticipated that with the need for maintaining physical distancing, reliable web-based assessments will be in demand for future frailty assessments.

Frailty assessments have traditionally relied on manual data entry and processing, which can be time-consuming and error-prone [21, 22]. Recent research has enabled computerized solutions. For example, an electronic frailty index calculation in the Electronic Medical Records (EMR) system has allowed for frailty measures in primary care for millions of older patients in the UK and several other European countries [23, 24]. More recently, a computerized frailty measure named the electronic frailty index (eFI) has been constructed based on the electronic Comprehensive Geriatric Assessment (eCGA, a multidisciplinary diagnostic assessment that evaluates many domains of older adults’ health and care needs) [25, 26]. Being available in the EMR system and as standalone software that runs on personal computers [25], the eFI-CGA is time-efficient and cost-effective. Even so, accessing the EMR from home can be cumbersome, and downloading/installing the standalone version upgrades can be inconvenient. This is especially true for health professionals who have no access to an EMR, including those who care for older adults in emergency, acute care, and long-term care settings.

Motivated to develop a web-based software tool for reliable frailty assessment, we conducted a thorough search and evaluation of the currently available web applications in the field. The purpose of this study is to provide insights to guide future web-based frailty assessment software development, including the web-based eFI-CGA. Our specific objectives were to: 1) understand what web applications exist for frailty assessment; 2) describe the usability of these applications; and 3) evaluate the challenges and opportunities of future web-based frailty assessments. Where applicable, the Preferred Reporting Items for Systematic Reviews and Meta-Analyses for Scoping Reviews guidelines (PRISMA-Scr) were applied [27] to allow a broad scope on the concept of web-based frailty assessments was obtained.

## Methods

### Information sources

**Fig. 1:**
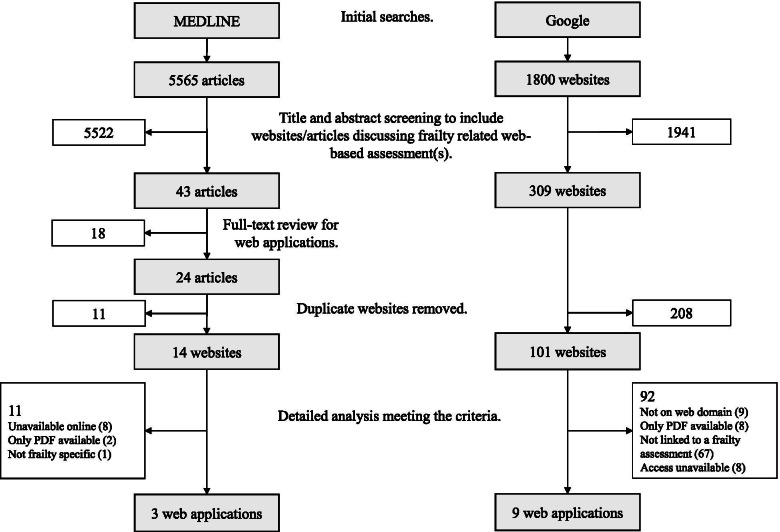
Flow diagram showing searches on Google and MEDLINE

The study used the electronic MEDLINE database, and the Google search engine. The MEDLINE-based literature search was conducted to extract information from peer-reviewed research literature. Additional databases (EMBASE, which captures more international publications, and CINAHL, which captures more publications aimed at nursing and allied health professions) were searched to verify the websites found, with broader coverage of the high-quality research from the searches. Considering the nature of web-based software applications, for which many developers do not publish journal articles on their web-based tools, a Google search was conducted in addition to the traditional literature search by directly identifying the websites linking to the software applications. Two researchers (RC and HL) conducted each of the searches independently, which were most recently updated on October 10, 2021 (Fig. 1).

### Search terms

The searches were performed based on three sets of keywords: Set 1: "online" or "web-based" or "website-based"; Set 2: "geriatric" or "frailty" or “older adult”; and Set 3: "assessment" or "software tool" or "application" or “calculator”. For the literature search, the three sets of keywords were combined with additional controlled vocabulary, which included “Internet”, “geriatrics”, “aged”, “frailty”, “mobile application”, “user-computer interface”, “geriatric assessment”, and “risk assessment” (Additional file 1). For the Google search, the individual terms in sets 1 through 3 were combined one on one, creating 36 unique search inputs (Additional file 2). A “*” sign was used to represent different suffixes of the same word in the search inputs/string where applicable.

### Search methods and selection of sources of evidence

The search strategy consisted of a standard literature search supplemented by a website search. The process was developed with the consultation and help of experienced librarians.

#### Literature search using MEDLINE

We included studies published from the inception of the database to October 10, 2021. Article titles and abstracts were screened to include publications discussing frailty assessment. Names of specific frailty related web-based assessments were then extracted from these relevant articles.

#### Website search using Google

To minimize the selection and customization impact of the Google search engine, all personal accounts were logged out of prior to each search, and the history, cookies, and cache were cleared. For each unique search input term, the first 50 non-advertisement results were retrieved, which sufficiently covered all relevant materials. This yielded a total of 1800 initial results (Fig. 1). All initial website titles and previews were sequentially screened to include websites related to frailty assessment. The resulting websites were then fully scanned to ensure they either hosted a web application on frailty assessment or included a direct external link to such a web application (Fig. 1).

An additional check of each of the individual web applications was conducted to ensure the assessment remains available online and meets the selection criteria as specified below. Finally, the results from these searches were combined, yielding a final list of web applications for further evaluation and analysis (Fig. 1).

### Selection criteria

Web-based frailty assessments were included if they met the following inclusion criteria: (1) related to frailty, (2) had a fillable form for data collection, (3) hosted on a web domain, (4) focused on older adults, and (5) interfaced in English. The exclusion criteria were: (1) not available online, (2) only displayed a non-fillable PDF version or conducted a calculation (i.e., a simple calculator that does not specify/assess any item), and (3) not accessible for research use or any independent application (e.g., assessments built into an EMR system or imbedded within a research project).

### Assessment characteristics selected and data charting process

Considering the common features of web applications, each application was evaluated applying IEEE recommendations for scoring that consisted of a comprehensive list of software criteria [28–34] of a total of 13 categories (Table 2). These included user friendly interface, effective data saving, completeness of health domain inclusion (Additional file 3), completeness of health item inclusion, the cost of usage, results interpretation availability, instructions and training availability, remote access, and conductance possibility (Table 2, top panel); and time efficiency of assessment, algorithm efficiency, security, environmental requirements, and browser requirements (Table 2, bottom panel). The categories were relevant to frailty and widespread application potential (e.g. promoting digital health and benefiting virtual health assessment) and reflected the populations, concepts, and contexts of interest in this review [27].

For each web application identified, an assessment score was assigned using a rating grade ranging from 0 to 3 for a given category, with higher scores meaning better performance (Table 2). For a category that could not be adequately evaluated (i.e. missed being reported by most websites), the category was not presented in the summed up score. The evaluation and scoring were conducted independently by two researchers (RC and HL). Any discrepancies were resolved through discussions between the researchers and consensuses were achieved upon the majority vote involving additional researchers (AM, XS).

## Results

### Characteristics of assessments

The final set of results consisted of twelve web-based frailty assessment applications, all of which were developed over the past six years (2015–2021). The majority of the web applications were provided by the USA (50%) or European countries (41%), with a focus on frailty grading and outcome prediction for specific patient groups (59%). Each of the web applications is summarized below and described with more details in Table 1.

**Table 1:** Web-based frailty assessments

Web-based Frailty Assessment Tool	Year^a^	Provided by^b^	Purpose of Tool^c^	Website URL^d^
**Edmonton Frail Scale**	2015	QxMD, USA	“A simple tool to assess frailty in older patients”	https://qxmd.com/calculate/calculator_595/edmonton-frail-scale
**Myeloma Frailty Score Calculator**	2015^e^	International Myeloma Working Group, collaborative effort involving USA, Canada, and European countries	“Predicts mortality and the risk of toxicity in elderly myeloma patients”	http://www.myelomafrailtyscorecalculator.net/Geriatric.aspx
**Johns Hopkins Frailty Assessment Calculator**	2016	Johns Hopkins, USA	“A standardized, evidenced-based method to assess frailty across clinical and research settings”	https://www.johnshopkinssolutions.com/solution/frailty/
**G8 Health Status Screening Tool**	2017^f^	Evidencio, Netherlands	“Identifies elderly cancer patients who would benefit from comprehensive geriatric assessment (CGA)”	https://www.evidencio.com/models/show/1045
**Liver Frailty Index**	2017^e^	University of California San Francisco, USA	“A tool specifically developed to objectively measure physical function in patients with cirrhosis”	https://liverfrailtyindex.ucsf.edu/
**QFrailty Risk Calculator**	2017	ClinRisk Ltd., England	“Works out your risk of developing Frailty”	https://qfrailty.org/
**Frailty Risk Calculator**	2018^e^	SmartData, University of Bologna, Italy	“Evaluates the probability of being either hospitalized or dying within next year for over 65 population living in Bologna”	http://smartdata.cs.unibo.it/frailtycalc/
**Senior Health Calculator**	2018	Beth Israel Deaconess Medical Center, USA	“Collects items of a comprehensive geriatric assessment (CGA) to calculate a frailty index (FI)”	https://www.bidmc.org/research/research-by-department/medicine/gerontology/calculator
**Modified Frailty Index**	2019^f^	Evidencio, Netherlands	“Evaluates risk of both morbidity and mortality in general surgery patients older than 60 years”	https://www.evidencio.com/models/show/1777
**MDS Specific Frailty Index**	2020	QxMD, USA	“Frailty scale specific to those patients with myelodysplastic syndrome that improves prognostication.”	https://qxmd.com/calculate/calculator_696/mds-specific-frailty-scale
**CIRS-G**	Not available from the original source	MDCalc, USA	“Quantifies the burden of disease in elderly patients (comorbidity scale)”	https://www.mdcalc.com/cumulative-illness-rating-scale-geriatric-cirs-g#use-cases
**Frailty Group Calculator**	2021	University of Oslo, Norway	“The frailty score (1 to 20) is calculated by multiplication of the weighted scores for… four geriatric assessment variables”	https://wide.shinyapps.io/app-frailty/

^a^Year the tool was created, unless otherwise indicated below

^b^Organization/company provided the web application, regardless the original assessment

^c^As described in the tool

^d^The webpage under the present evaluation

^e^ Publication date of the corresponding article

^f^ Date of last revision

Released in 2015, the QxMD provided web *Edmonton Frail Scale* consists of 11 items over multiple health domains. It provides a simple way to assess frailty in older adults and can be completed in 5 min on average.

Also released in 2015, the *Myeloma Frailty Score Calculator* aids in the prognosis of elderly myeloma patients, through assessing 31 items on age, comorbidities, cognitive, and physical functions, which can be saved in a PDF document along with the calculated score.

In the following year, *the Johns Hopkins Frailty Assessment Calculator* was released to assess the five-item frailty phenotype [8]. A free trial is limited to 5 calculations; unlimited calculations and guidebook and database access can be obtained with an annual subscription.

*The Geriatric 8 (G8) Health Status Screening Tool* was updated in 2017, for identifying older cancer patients who may benefit from a CGA through assessing physical and neuropsychological health. Free accounts can download a PDF assessment; a subscription leads to unlimited downloads and the ability to add notes to the PDF.

*The Liver Frailty Index* was also released in 2017 to assess physical frailty in patients with chronic liver disease and/or cirrhosis. It assesses three performance-based items: grip strength, chair stands, and balance, with detailed instructions including diagrams for use.

Also released in 2017, *the QFrailty Risk Calculator* assesses older adults’ risk of developing frailty involving 40+ deficits over 10 health domains. The software estimates the frailty degrees (e.g “mild” or “severe”), and the two-year hospitalization and death risks.

*The Frailty Risk Calculator* was released in 2018. Utilizing social and clinical variables, this tool estimates the probability of hospitalization or death within the next year for older adults.

Also released in 2018, *the Senior Health Calculator* uses the CGA items to produce a deficit accumulation based FI. Fifty items on medical history, functional status, performance tests, and nutritional status are assessed, and FI calculation can be based only on the first two domains. The input data, FI, and summary may be saved or printed as PDF.

*The Modified Frailty Index* was recently updated in 2019 and assesses the morbidity and mortality risks in older general surgery patients based on the FI. Free accounts can download a PDF assessment; a subscription leads to unlimited downloads and adding notes to the PDF.

*The Myelodysplastic syndromes (MDS) Specific Frailty Index* was released in 2020 and evaluates frailty in patients with myelodysplastic syndrome. Seventeen items are included to calculate a frailty scale ratio, composite score, and estimated survival outcome. Users may manually copy/paste the input and output to the local computer.

*The Cumulative Illness Rating Scale-Geriatric (CIRS-G)* is an assessment that quantifies the disease burden in older adults. It uses 14 multiple-choice comorbidities to produce a frailty score. With a free account, users can copy assessment inputs and results to the local computer.

In 2021, the *Frailty Group Calculator* was released. It assesses 21 items from the Charlson Comorbidity Index, Geriatric Nutritional Risk Index, and activities of daily living to produce a frailty score. The website includes a detailed description of all terms used (Table 1).

### Synthesis of results

**Fig. 2:**
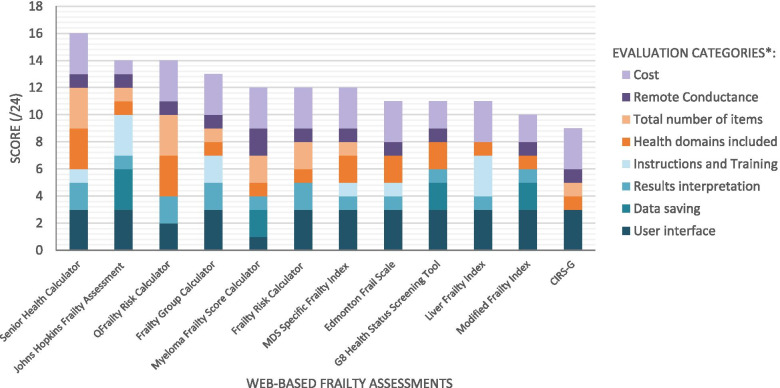
Graph comparing the total scores of each assessment. *The scoring was based on the categories as given in the legends. Additional categories (time efficiency, security, algorithm efficiency, environmental and browser requirements) could not be scored due to limited information from the applications under evaluation

Eight feature categories could be applied to scoring these web applications (i.e., assigning a 0 through 3 to each category), making 24 the maximum possible sum-up score that a web application could receive for high performance (Table 2). Figure 2 shows the categorical and sum-up scores of each web application. The two categories that scored with high values among the applications were User Interface and Cost (2.75/3 and 2.67/3, respectively), whereas the other categories had a mean score of 1.67 or lower (Fig. 2). The category with the lowest score was Data Saving, with only 4 assessments not scoring a 0, as most of the web applications permitted no or very primitive data saving or data loading.

**Table 2:** Evaluation categories and criteria

Score Category^a^	0	1	2	3
**User Interface**	Unreadable or unfillable	Poor readability, poor selection choices	Good readability, poor selection choices OR poor readability, good selection choices	Good readability, good selection choices
**Data Saving**	Unable to save filled assessment form or results	Able to save assessment results only	Able to save filled assessment form and results	Able to save filled assessment form and results, and reload assessments for edits
**Number of Health Domains**	< 3 health domains included	3–6 health domains included	7–10 health domains included	> 10 health domains included.
**Number of Assessment Items**	< 14 items included	14–25 items included	26–37 items included	> 37 items included
**Cost**	Available for a fee or with subscription purchase	Available with a free trial	Available for free but payment required to access certain features	Available for free or with a free account
**Results Interpretation**	No interpretation or explanation of results provided	Brief interpretation of results provided (< 5 words)	Sufficient interpretation	Detailed interpretation and/or additional information provided
**Instructions and Training**	No instructions or training provided	Minimal instructions provided within assessment	Instruction manual or other training resources provided (e.g., video tutorial)	Instructions provided within assessment along with additional training or instructional resources
**Remote Conductance**	Cannot be completed virtually (i.e. includes mandatory items that cannot be completed remotely)	Can be completed virtually with alterations (i.e. items that cannot be completed remotely are not mandatory)	Can be completed virtually through video call (i.e. assessment includes items that require a video call)	Can be completed virtually through phone call (i.e. assessment does not include items that require a video call)
**Time Efficiency**	Not reported	> 30 min on average	20–30 min on average	< 20 min on average
**Algorithm Efficiency**	Not reported	Polynomial runtime complexity (≥ O(N^3^), O(N^4^), etc.)	Quadratic runtime complexity (O(N^2^))	Linear or logarithmic runtime complexity (O(N) or O(log N))
**Security**	No login/account security, data access security, or server security	Includes one of: login/account security, data access security, or server security	Includes two of: login/account security, data access security, or server security	Includes login/account security, data access security, and server security
**Environmental Requirements**	Dependant on multiple third-party applications; sophisticated hardware/software tools required to use the application (e.g. latest model of iPhone/iPad, minimum 8 GB of RAM)	Few (< 3) external third-party applications are required for the tool operate; software is platform specific (e.g. only works on Windows/MAC/iOS/etc.)	External software dependencies are packaged and installed with the assessment tool; software runs on all desktop operating systems or all smartphones (e.g. is available on Android and iOS)	No external software dependencies exist for the software; software can be accessed and operated on all digital platforms.
**Browser Requirements**	Compatible with minimum browsers (1).	Compatible with limited browsers (2)	Compatible with more browsers. (3–4)	Compatible with most major browsers (≥5)

^a^ The scoring was based on the top portion of the categories above the bolded line

## Discussion

We conducted this study to understand what frailty assessment tools are currently available online and to evaluate their usability considering a large number of feature categories. To the best of our knowledge, this is the first attempt to identify and summarize web-based frailty assessment applications in a scoping review. This is also the first known study that comprehensively evaluates the applicability and limitations of web applications. The research has allowed us to generate important insights into successful future development of online software tools in support of early detection and management of frailty.

The assessments under evaluation showed several essential merits and have multiple advantages. Most applications have implemented a highly friendly user interface. One crucial benefit of having a web-based assessment is the convenience and ease of completing the assessment with simple selections and mouse clicks [20, 23]. For example, the web applications realized user interface functionality by employing radio button selection for the binary “yes or no” questions, which is more time conservative than using a drop-down menu or text box. Most applications also appeared to be highly cost efficient. Making assessments available for free can help maximize the potential for adoption and impact. Having easily accessible frailty assessments will encourage use and scale up the effort of frailty management.

Despite these beneficial qualities, several important areas may be improved in future development of web-based frailty tools. For example, the assessment pages often lacked clear and comprehensive instructions or training materials. Including sufficient materials of training and education can be crucial for potential users to accurately and effectively complete the assessments, especially regarding the specifics of some performance items (e.g. specific version of the “sit to stand” test). Similar arguments can apply to including helpful materials for results interpretation. Even though an assessment can yield a score or a frailty rating, these numbers would need interpretation with context. Adding this information can help the user understand the assessment outcome more accurately, benefiting patient care planning.

It is also worth noting that the existing web applications commonly included limited options for data saving and retrieval. Although copying of the completed assessment and the associated scoring might be allowed by some, working with the data or even reloading the saved assessments for processing/editing were typically unmanageable. In the context of virtual health appointments, this feature is needed in scenarios where an assessment must be completed over more than one session due to time restrictions, interrupted Internet connections, or other disruptions that healthcare professionals may experience [35].

Further, a majority of the assessments considered only a limited number of health domains and/or total items. Because frailty is commonly recognized as a multidimensional syndrome characterized by the loss of physiological reserve in multiple health systems [6, 9] frailty assessments can have improved precision relating the outcomes when a wide range of health domains and items are considered, for a comprehensive overview of patients’ health. For example, it has been recommended that a deficit accumulation-based frailty index includes no less than 30 individual items when possible [36, 37].

Our study has several limitations. The literature search largely used the MEDLINE databases. Although applied a thorough search strategy was applied and checked against additional databases to increase coverage of high quality research, the literature searches yielded only a limited number of the websites. This situation is determined by the general lack of research publications for web applications on frailty. When supplemented by the Google search, more websites hosting such web applications were retrieved directly, regardless of whetherif any scientific publications were associated with them. While the search strategy has properly targeted the research topic at hand, it is still possible that some web applications that are only available from less prominent resources may have been overlooked.

Likewise, frailty assessments that are electronic but not web-based, such as the EMR data analyses of frailty within the UK and several other countries [23, 38], and the frailty assessment within the EMRs [26], were not included. Other useful but non web-based assessments that may have been excluded can include those that are eimbedded in specific clinical programs or research investigations such as InterRAI, a well-established global initiative, in which a wide-range of embedded functional tools are developed, including frailty scales [39]. As program-imbedded tools are not readily identifiable or accessible via Internet domains, they are not always searchable following a consistent approach (e.g., the one described in the methodology), and applying prior knowledge about these embedded tools in the search would likely lead to certain additions, but in the meantime, inconsistent inclusions.

Also, while using an online search engine to supplement the standard literature search has merit for a more adequate website identification, it is acknowledged that the commercial aspect of a search engine can potentially compromise an unbiased search. In addressing this limitation, our strategy has allowed the exclusion of all results labeled as advertisements, the clean-up of all personal accounts, and the scanning of a sufficient number of results, avoiding the possibility of limiting the search to retrieving only to the “most popular” results displayed first.

Further, as the study focused is the availability, accessibility, functionality, and usability of the web applications, we have included all frailty conceptualization and operationalization approaches found; i.e., the content validity and psychometrics of the frailty assessments themselves based upon the web applications developed were not considerations for exclusion [40]. Also, several categories of the evaluation (time efficiency, security, algorithm efficiency, environmental requirements, and browser requirements) were not assessable, due to the lack of data provided by the web applications under review. Information on these aspects can be fundamental in software appraisal [25, 28, 30–34], and we encourage future developments to take these into consideration with software implementation and report how these aspects are addressed.

Despite these limitations, our study contributes to the research field and is relevant to advancing early detection and management of frailty. We have applied the established software evaluation approaches [28–34] for an insightful understanding of the available web applications, highlighting the important features to consider for inclusion in future development, including our ongoing effort in advancing a fully functional web-based eFI-CGA (https://efi-cga.ca/).

Creating robust web-based frailty assessments presents opportunities for more widely accessible frailty monitoring and management. In this regard, standalone software tools are limited by the need to manually download and install software and any needed updates. Standalone applications can also restrict effective data sharing, as data is only saved locally on the user’s computer. Similarly, assessments embedded in EMR systems are only accessible from certain locations (e.g., family physician clinics), restricting accessibility by health professionals who care for older adults in acute and long-term settings, and limiting individualized integrated care. Web frailty assessments may allow for a much-required continuity of care for patients, particularly for frail older adults with complex medical needs, as shown by the ability to predict hospital outcomes from frailty measures [18–21, 41–43].

Another major advantage of web-based frailty assessments is the potential of increasing the efficiency of data management and large-scale information sharing. In Canada, and perhaps in some other countries, the lack of interoperability of EMR systems represents a major barrier to a unified electronic health solution [44]. Taking the EMR based eFI-CGA as an example, vendors of different EMR systems have automated the assessment in different ways, making it difficult to integrate measurements and outputs [25]. A web application can provide a solution through the universally accessible Internet, which is meaningful for promoting efforts in providing better care for people in the aging society.

The COVID-19 pandemic and its consequences have also highlighted the value of web-based assessments, with healthcare providers needing flexible health assessments to accommodate physical distancing restrictions [15]. For instance, we have seen an increasing number of requests since the beginning of the pandemic for obtaining a copy of the standalone eFI-CGA software tool [25]. Having web-based assessments would allow for frailty management to continue during times of limited contact, as they can be readily accessed.

One concern related to virtual health appointments may be the ability of older adults to use the necessary technologies such as the Internet or video call platforms. However, throughout the course of the pandemic, many patients or providers who may have been deemed as “technologically limited” have adapted and become proficient in using video calls for other activities such as exercise, socializing, and learning [43]. While an initial learning curve is expected, there is a generally positive perspective towards virtual care applying computerized technologies within the new generation of older adults [45].

A foreseeable challenge to widespread adoption of web-based frailty assessments is the patient privacy and data security concerns. Given that there are many healthcare systems already using external data platforms including therapy delivery and patient monitoring, it is anticipated that with sufficient precautions and regulations, perhaps one day the same can be done for frailty. Even though previous efforts to expand virtual care met with some resistance [43], the COVID-19 pandemic has “kickstarted” this expansion. As different aspects of virtual healthcare advance, it is important that frailty assessments advance concurrently – if we expect frailty identification and management to have a more central role in older adults’ healthcare, as recommended [10, 46], we must ensure it evolves as the healthcare system does. The process may take time and effort, and the present study aims to aid the transition by highlighting the currently available web-based frailty assessments and providing insights on developing web applications for use in virtual care.

## Conclusions

Taken together, web-based applications represent a viable option for remote frailty assessments and multidisciplinary integrated care of older adults. Presented following the PRISMA-ScR, this scoping review has shown that despite the availability of web-based frailty assessments on the Internet, many lack certain features needed for professional use in healthcare settings. This situation calls for fully comprehensive web-based applications, taking into account a number of key functions linking the front and back ends and paying special attention to secure data management. It is anticipated that having reliable and effective web-based health applications will promote remote assessments as an aspect of virtual care as common practice in the future.

## Supplementary Information

**Additional file 1.** Search terms and controlled vocabulary for the literature search using the electronic databases.

**Additional file 2.** Google Search Strings for Websites.

**Additional file 3.** Health domains and examples.

**Additional file 4.** Web-based applications and associated scores assigned during analysis.

## Data Availability

All data (i.e., the websites) analysed during this study are included in this published article (Table 1). An additional data file listing the original data (i.e., the websites) and the associated scores assigned for the analysis is also provided (Additional file 4).

## Abbreviations

COVID-19: Coronavirus disease 2019
eCGA: Electronic comprehensive geriatric assessment
FI: Frailty index
eFI-CGA: Electronic frailty index comprehensive geriatric assessment
EMR: Electronic medical record
G8: Geriatric 8
MDS: Myelodysplastic syndromes (MDS)
CIRS-G: Cumulative Illness Rating Scale-Geriatric

## References

[1] Vaupel JW, Manton KG, Stallard E (1979). The impact of heterogeneity in individual frailty on the dynamics of mortality. Demography..

[2] Clegg A, Young J, Iliffe S, Rikkert MO, Rockwood K (2013). Frailty in elderly people. Lancet (London, England).

[3] Sternberg SA, Wershof Schwartz A, Karunananthan S, Bergman H, Mark CA (2011). The identification of frailty: a systematic literature review. J Am Geriatr Soc.

[4] Pijpers E, Ferreira I, Stehouwer CD, Nieuwenhuijzen Kruseman AC (2012). The frailty dilemma. Review of the predictive accuracy of major frailty scores. Eur J Intern Med.

[5] Morley JE, Vellas B, van Kan GA, Anker SD, Bauer JM, Bernabei R (2013). Frailty consensus: a call to action. J Am Med Dir Assoc.

[6] Mitnitski A, Song X, Rockwood K (2013). Assessing biological aging: the origin of deficit accumulation. Biogerontology..

[7] Fried LP, Walston J, Hazzard W (1998). Frailty and failure to thrive. Principles of geriatric medicine and gerontology.

[8] Fried LP, Tangen CM, Walston J, Newman AB, Hirsch C, Gottdiener J (2001). Frailty in older adults: evidence for a phenotype. J Gerontol A Biol Sci Med Sci.

[9] Rockwood K, Mitnitski A (2011). Frailty defined by deficit accumulation and geriatric medicine defined by frailty. Clin Geriatr Med.

[10] Walston J, Bandeen-Roche K, Buta B, Bergman H, Gill TM, Morley JE (2019). Moving Frailty Toward Clinical Practice: NIA Intramural Frailty Science Symposium Summary. J Am Geriatr Soc.

[11] Zhang XM, Jiao J, Cao J, Huo XP, Zhu C, Wu XJ (2021). Frailty as a predictor of mortality among patients with COVID-19: a systematic review and meta-analysis. BMC Geriatr.

[12] Neumann-Podczaska A, Al-Saad SR, Karbowski LM, Chojnicki M, Tobis S, Wieczorowska-Tobis K (2020). COVID 19 - clinical picture in the elderly population: a qualitative systematic review. Aging Dis.

[13] Hewwit J, Carter B, Vilches-Moraga A, Quinn TJ, Braude P, Verduri A (2020). The effect of frailty on survival in patients with COVID-19 (COPE): a multicentre, European, observational cohort study. Lancet Public Health.

[14] Wosik J, Fudim M, Cameron B, Gellad ZF, Cho A, Phinney D (2020). Telehealth transformation: COVID-19 and the rise of virtual care. J Am Med Inform Assoc.

[15] Golinelli D, Boetto E, Carullo G, Nuzzolese AG, Landini MP, Fantini MP (2020). Adoption of digital Technologies in Health Care during the COVID-19 pandemic: systematic review of early scientific literature. J Med Internet Res.

[16] Wolfe MK, McDonald NC, Holmes GM (2020). Transportation barriers to health Care in the United States: findings from the National Health Interview Survey, 1997–2017. Am J Public Health.

[17] Syed ST, Gerber BS, Sharp LK (2013). Traveling towards disease: transportation barriers to health care access. J Community Health.

[18] Bensley RJ, Lewis JB (2002). Analysis of internet-based health assessments. Health Promot Pract.

[19] Gray L, Vincent R, Martin-Khan M, Varghese P, Wootton R (2006). Processing time for an online geriatric assessment tool. J Telemed Telecare.

[20] Bot AG, Menendez ME, Neuhaus V, Mudgal CS, Ring D (2013). The comparison of paper- and web-based questionnaires in patients with hand and upper extremity illness. Hand (NY).

[21] Newgard CD, Zive D, Jui J, Weathers C, Daya M (2012). Electronic versus manual data processing: evaluating the use of electronic health Records in out-of-hospital Clinical Research. Acad Emerg Med.

[22] Pavlovic I, Kern T, Miklavcic D (2009). Comparison of paper-based and electronic data collection process in clinical trials: costs simulation study. Contemp Clin Trials.

[23] Clegg A, Bates C, Young J, Ryan R, Nichols L, Ann Teale E (2013). Development and validation of an electronic frailty index using routine primary care electronic health record data. Age Ageing.

[24] Devereux N, Ellis G, Dobie L, Baughan P, Monaghan T (2019). Testing a proactive approach to frailty identification: the electronic frailty index. BMJ Open Qual.

[25] Sepehri K, Braley MS, Chinda B, Zou M, Tang B, Park G (2020). A computerized frailty assessment tool at points-of-care: development of a standalone electronic comprehensive geriatric assessment/frailty index (eFI-CGA). Front Public Health.

[26] Garm A, Park GH, Song X (2017). Using an electronic comprehensive geriatric assessment and health coaching to prevent frailty in primary care: the CARES model. Med Clin Rev.

[27] Tricco AC, Lillie E, Zarin W, O'Brien KK, Colquhoun H, Levac D (2018). PRISMA extension for scoping reviews (PRISMA-ScR): checklist and explanation. Ann Intern Med.

[28] Yeratziotis A, Van Greunen D, Pottas. (2011). Recommendations for usable security in online health social networks. 2011 6th International Conference on Pervasive Computing and Applications.

[29] Galitz OW (2007). The essential guide to user Interface design: an introduction to GUI design principles and techniques.

[30] Calic T, Dascalu S, Egbert D (2008). Tools for MDA software development: evaluation criteria and set of desirable features. Fifth International Conference on Information Technology: New Generations (itng 2008).

[31] Anand V, Saxena D (2013). Comparative study of modern web browsers based on their performance and evolution. 2013 IEEE International Conference on Computational Intelligence and Computing Research.

[32] Kaur A, Dani D, Agrawal G (2017). Evaluating the accessibility, usability and security of hospitals websites: an exploratory study. 2017 7th International Conference on Cloud Computing, Data Science & Engineering-Confluence.

[33] Yangqing Z, Hui Y, Hua L, Lianming Z. Design of a new web database security model. In: 2009 Second international symposium on electronic commerce and security, vol. 2009, 1: IEEE; 2009. p. 292–5.

[34] ISO/IEC JTC 1/SC 7 Technical Committee. ISO/IEC/IEEE International Standard - Systems and software engineering - Engineering and management of websites for systems, software, and services information. In: ISO/IEC/IEEE 23026 First edition. IEEE; 2015. p. 1–54.

[35] Khurshid Z, De Brún A, Moore G, McAuliffe E (2020). Virtual adaptation of traditional healthcare quality improvement training in response to COVID-19: a rapid narrative review. Hum Resour Health.

[36] Song X, Mitnitski A, Rockwood K (2014). Age-related deficit accumulation and the risk of late-life dementia. Alzheimers Res Ther.

[37] Searle SD, Mitnitski A, Gahbauer EA, Gill TM, Rockwood K (2008). A standard procedure for creating a frailty index. BMC Geriatr.

[38] Abbasi M, Khera S, Dabravolskaj J, Vandermeer B, Theou O, Rolfson D (2019). A cross-sectional study examining convergent validity of a frailty index based on electronic medical records in a Canadian primary care program. BMC Geriatr.

[39] Morris JN, Howard EP, Steel KR (2016). Development of the interRAI home care frailty scale. BMC Geriatr.

[40] Broad A, Carter B, Mckelvie S, Hewitt J (2020). The convergent validity of the electronic frailty index (eFI) with the clinical frailty scale (CFS). Geriatrics (Basel).

[41] Kerminen H, Huhtala H, Jäntti P (2020). Frailty index and functional level upon admission predict hospital outcomes: an interRAI-based cohort study of older patients in post-acute care hospitals. BMC Geriatr.

[42] Rockwood K, Mitnitski A, Song X, Steen B, Skoog I (2006). Long-term risks of death and institutionalization of elderly people in relation to deficit accumulation at age 70. J Am Geriatr Soc.

[43] Schwamm LH, Estrada J, Erskine A, Licurse A (2020). Virtual care: new models of caring for our patients and workforce. Lancet Digit Health.

[44] Chang F, Gupta N (2015). Progress in electronic medical record adoption in Canada. Can Fam Physician.

[45] McDonald AP, Rizzotti R, Rivera JM, D'Arcy RCN, Park G, Song X (2021). Toward improved homecare of frail older adults: a focus group study synthesizing patient and caregiver perspectives. Aging Med.

[46] Mehta P, Lemon G, Hight L, Allan A, Li C, Pandher SK (2021). A systematic review of clinical practice guidelines for identification and management of frailty. J Nutr Health Aging.

