# Supplementary material for: Web-based software applications for frailty assessment in older adults: a scoping review of current status with insights into future development
Source: BMC Geriatr. 2021 Dec 18;21:723. doi: 10.1186/s12877-021-02660-6 (PMC8683817; doi:10.1186/s12877-021-02660-6)
Supplement: Supplementary file 1 — Additional file 1. Search terms and controlled vocabulary for the literature search using the electronic databases. [file 12877_2021_2660_MOESM1_ESM.docx]

**Appendix 1.** Search terms and controlled vocabulary for the literature search using the electronic databases.

1. exp Internet/
2. (online OR web based OR website).ti,ab.
3. OR/1,2
4. Geriatrics/
5. exp Aged/
6. Frailty/
7. (geriatric* OR older adult* OR frail*).ti,ab.
8. OR/4-7
9. Mobile applications/
10. User-computer interface/
11. Geriatric assessment/
12. Risk assessment/
13. (assessment OR tool OR application OR calculator).ti,ab.
14. OR/9-13

AND/3,8,14
