# Supplementary material for: Web-based software applications for frailty assessment in older adults: a scoping review of current status with insights into future development
Source: BMC Geriatr. 2021 Dec 18;21:723. doi: 10.1186/s12877-021-02660-6 (PMC8683817; doi:10.1186/s12877-021-02660-6)
Supplement: Supplementary file 2 — Additional file 2. Google Search Strings for Websites. [file 12877_2021_2660_MOESM2_ESM.docx]

**Appendix 2.** Google Search Strings for Websites.

1. Online geriatric assessment
2. Online geriatric software tool
3. Online geriatric application
4. Online geriatric calculator
5. Online frailty assessment
6. Online frailty software tool
7. Online frailty application
8. Online frailty calculator
9. Online older adult assessment
10. Online older adult software tool
11. Online older adult application
12. Online older adult calculator
13. Web-based geriatric assessment
14. Web-based geriatric software tool
15. Web-based geriatric application
16. Web-based geriatric calculator
17. Web-based frailty assessment
18. Web-based frailty software tool
19. Web-based frailty application
20. Web-based frailty calculator
21. Web-based older adult assessment
22. Web-based older adult software tool
23. Web-based older adult application
24. Web-based older adult calculator
25. Website-based geriatric assessment
26. Website-based geriatric software tool
27. Website-based geriatric application
28. Website-based geriatric calculator
29. Website-based frailty assessment
30. Website-based frailty software tool
31. Website-based frailty application
32. Website-based frailty calculator
33. Website-based older adult assessment
34. Website-based older adult software tool
35. Website-based older adult application
36. Website-based older adult calculator
