# Supplementary material for: Web-based software applications for frailty assessment in older adults: a scoping review of current status with insights into future development
Source: BMC Geriatr. 2021 Dec 18;21:723. doi: 10.1186/s12877-021-02660-6 (PMC8683817; doi:10.1186/s12877-021-02660-6)
Supplement: Supplementary file 3 — Additional file 3. Health domains and examples. [file 12877_2021_2660_MOESM3_ESM.docx]

**Appendix 3 :** Health domains and examples.

| Health Domain | Example(s) |
| --- | --- |
| Lifestyle | Alcohol, smoking, living alone, marital status |
| Sensory conditions | Blindness, deafness |
| Neurological conditions | Epilepsy |
| Physical health conditions | Cancer, asthma, arthritis |
| Symptoms | Pain |
| Cognition | Dementia, MMSE |
| Mental health | Mood, depression |
| Strength | Dominant handgrip strength |
| Balance | Five repeated chair stands, falls |
| Mobility | Gate speed |
| Exercise | Frequency of exercise |
| Nutrition | Appetite, BMI |
| ADLs | Bathing, dressing |
| IADLs | Shopping, transportation |
| Exhaustion/fatigue | Global Fatigue Score |
| Elimination | Continence |
| Medication | Number of medications used |
| Health service use | Number of hospital/ER visits |
| General health | Patient rates their overall health |
| Laboratory values | WBC, Creatinine clearance |
| Demographics | Age, gender |
