# Supplementary material for: Web-based software applications for frailty assessment in older adults: a scoping review of current status with insights into future development
Source: BMC Geriatr. 2021 Dec 18;21:723. doi: 10.1186/s12877-021-02660-6 (PMC8683817; doi:10.1186/s12877-021-02660-6)
Supplement: Supplementary file 4 — Additional file 4. Web-based applications and associated scores assigned during analysis. [file 12877_2021_2660_MOESM4_ESM.docx]

**Appendix 4.** Web-based applications and associated scores assigned during analysis.

| **Category**  **Application URL** | **User interface** | **Data saving** | **Results interpretation** | **Instructions and Training** | **Health domains included** | **Total number of items** | **Remote Conductance** | **Cost** |
| --- | --- | --- | --- | --- | --- | --- | --- | --- |
| **Senior Health Calculator:** https://www.bidmc.org/research/research-by-department/medicine/gerontology/calculator | 3 | 0 | 2 | 1 | 3 | 3 | 1 | 3 |
| **Johns Hopkins Frailty Assessment Calculator:**  https://www.johnshopkinssolutions.com/solution/frailty/ | 3 | 3 | 1 | 3 | 1 | 1 | 1 | 1 |
| **QFrailty Risk Calculator:**  https://qfrailty.org/ | 2 | 0 | 2 | 0 | 3 | 3 | 1 | 3 |
| **Myeloma Frailty Score Calculator:**  http://www.myelomafrailtyscorecalculator.net/Geriatric.aspx | 1 | 2 | 1 | 0 | 1 | 2 | 2 | 3 |
| **Frailty Risk Calculator:**  http://smartdata.cs.unibo.it/frailtycalc/ | 3 | 0 | 2 | 0 | 1 | 2 | 1 | 3 |
| **MDS Specific Frailty Index:**  https://qxmd.com/calculate/calculator_696/mds-specific-frailty-scale | 3 | 0 | 1 | 1 | 2 | 1 | 1 | 3 |
| **Edmonton Frail Scale:**  https://qxmd.com/calculate/calculator_595/edmonton-frail-scale | 3 | 0 | 1 | 1 | 2 | 0 | 1 | 3 |
| **G8 Health Status Screening Tool:**  https://www.evidencio.com/models/show/1045 | 3 | 2 | 1 | 0 | 2 | 0 | 1 | 2 |
| **Liver Frailty Index:**  https://liverfrailtyindex.ucsf.edu/ | 3 | 0 | 1 | 3 | 1 | 0 | 0 | 3 |
| **Modified Frailty Index:**  https://www.evidencio.com/models/show/1777 | 3 | 2 | 1 | 0 | 1 | 0 | 1 | 2 |
| **CIRS-G:**  https://www.mdcalc.com/cumulative-illness-rating-scale-geriatric-cirs-g#use-cases | 3 | 0 | 0 | 0 | 1 | 1 | 1 | 3 |
| **Frailty Group Calculator:**  https://wide.shinyapps.io/app-frailty/ | 3 | 0 | 2 | 2 | 1 | 1 | 1 | 3 |
